# Inflammatory Biomarkers and Clinical Outcomes in Hospitalized Patients with COVID-19 and Pre-Existing Heart Failure: A Single-Center Cohort Study

**DOI:** 10.3390/jcm15062209

**Published:** 2026-03-13

**Authors:** Maria-Laura Craciun, Adina Cristiana Avram, Ana-Maria Pah, Cristina Vacarescu, Diana-Maria Mateescu, Adrian Cosmin Ilie, Ioana Georgiana Cotet, Claudia Raluca Balasa Virzob, Simina Crisan, Claudiu Avram, Florina Buleu, Daian Ionel Popa, Zorin Petrisor Crainiceanu, Stela Iurciuc

**Affiliations:** 1 Cardiology Department, “Victor Babes” University of Medicine and Pharmacy, Eftimie Murgu Square 2, 300041 Timisoara, Romania; laura.craciun@umft.ro (M.-L.C.); simina.crisan@umft.ro (S.C.); iurciuc.stela@umft.ro (S.I.); 2 Department of Internal Medicine I, “Victor Babes” University of Medicine and Pharmacy, Eftimie Murgu Square 2, 300041 Timisoara, Romania; avram.adina@umft.ro; 3 Department of General Medicine, Doctoral School, “Victor Babes” University of Medicine and Pharmacy, Eftimie Murgu Square 2, 300041 Timisoara, Romania; diana.mateescu@umft.ro (D.-M.M.); ioana.cotet@umft.ro (I.G.C.); 4 Department of Public Health and Sanitary Management, “Victor Babes” University of Medicine and Pharmacy, Eftimie Murgu Square 2, 300041 Timisoara, Romania; ilie.adrian@umft.ro; 5 Centre for Translational Research and Systems Medicine, Faculty of Medicine, “Victor Babes” University of Medicine and Pharmacy, Eftimie Murgu Square 2, 300041 Timisoara, Romania; 6 Department of Clinic Nursing, “Victor Babes” University of Medicine and Pharmacy, Eftimie Murgu Square 2, 300041 Timisoara, Romania; virzob.claudia@umft.ro; 7 Department XVI, Balneology, Medical Recovery and Rheumatology, “Victor Babes” University of Medicine and Pharmacy, Eftimie Murgu Square 2, 300041 Timisoara, Romania; avram.claudiu@umft.ro; 8 Department VI, Discipline of Internal Medicine and Ambulatory Care, Prevention and Cardiovascular Recovery, Faculty of Medicine, “Victor Babes” University of Medicine and Pharmacy, Eftimie Murgu Square 2, 300041 Timisoara, Romania; florina.buleu@umft.ro; 9 Research Center for Medical Communication, “Victor Babes” University of Medicine and Pharmacy, Eftimie Murgu Square 2, 300041 Timisoara, Romania; daian-ionel.popa@umft.ro; 10 Plastic Surgery Department, “Victor Babes” University of Medicine and Pharmacy, Eftimie Murgu Square 2, 300041 Timisoara, Romania; crainiceanu.zorin@umft.ro

**Keywords:** heart failure, COVID-19, interleukin-6, inflammatory biomarkers, systemic inflammation, sepsis, mortality, risk stratification, cardiovascular disease, hospitalization

## Abstract

**Background/Objectives**: Patients with pre-existing heart failure (HF) represent a clinically vulnerable population with increased susceptibility to adverse outcomes during acute systemic illnesses, including coronavirus disease 2019 (COVID-19). Systemic inflammation is increasingly recognized as a central pathophysiological mechanism linking cardiovascular vulnerability with infection-related organ dysfunction. However, the prognostic role of inflammatory biomarkers in hospitalized COVID-19 patients with pre-existing HF remains incompletely defined. This study aimed to evaluate the association between inflammatory biomarkers and clinical outcomes in this high-risk population. **Methods**: This retrospective single-center cohort study included 395 consecutive adult patients hospitalized with confirmed COVID-19 between March 2020 and December 2024 at a tertiary referral center. Pre-existing HF was documented in 143 patients (36.2%). Inflammatory biomarkers, including C-reactive protein (CRP), interleukin-6 (IL-6), procalcitonin, and D-dimer, were measured at admission. The primary outcomes were development of sepsis and in-hospital mortality. Multivariable logistic regression models were constructed to identify independent predictors of adverse outcomes after adjustment for demographic characteristics, comorbidities, disease severity, and cardiac biomarkers. **Results**: Patients with pre-existing HF had significantly higher in-hospital mortality compared with those without HF (11.9% vs. 4.8%, *p* = 0.016) and showed a trend toward increased intensive care unit admission. HF patients exhibited higher admission IL-6 levels, indicating enhanced inflammatory activation. In univariable analysis, HF was associated with mortality (OR 2.67, 95% CI 1.22–5.83, *p* = 0.014). After multivariable adjustment, the association between HF and mortality was attenuated, whereas IL-6 remained an independent predictor of mortality (adjusted OR 1.38, 95% CI 1.04–1.82, *p* = 0.021). Elevated IL-6 and procalcitonin levels were also independently associated with sepsis development. **Conclusions**: Pre-existing heart failure identifies a population at increased risk of adverse outcomes in hospitalized COVID-19 patients, and this excess risk appears to be partly mediated by systemic inflammatory activation. Interleukin-6 emerged as a key biomarker linking cardiovascular vulnerability, immune dysregulation, and clinical deterioration. These findings support the potential role of inflammation-based risk stratification to improve prognostic assessment and guide personalized management in high-risk patients with underlying cardiovascular disease.

## 1. Introduction

Heart failure (HF) remains a leading cause of morbidity, mortality, and healthcare utilization worldwide, with a continuously rising prevalence driven by population aging and improved survival after major cardiovascular events. Contemporary epidemiologic estimates indicate that HF affects >64 million people globally, underscoring its major public health and economic burden. Management is phenotype-specific (HFrEF/HFmrEF/HFpEF; acute vs. chronic presentations) and relies on guideline-directed evaluation and therapy, yet clinical outcomes remain heterogeneous, particularly in hospitalized patients who frequently carry a high comorbidity load and experience recurrent decompensations [1,2,3].

Beyond hemodynamic impairment and neurohormonal activation, HF is increasingly recognized as a systemic inflammatory syndrome, in which persistent, low-grade inflammation contributes to myocardial remodeling, endothelial dysfunction, multiorgan injury, skeletal muscle wasting, and progressive clinical deterioration. Mechanistically, innate and adaptive immune activation can be triggered by tissue injury, congestion-related organ hypoperfusion, comorbidities (e.g., obesity, diabetes, chronic kidney disease), and inflammatory signaling within the myocardium. These pathways promote fibrosis, cardiomyocyte dysfunction, altered calcium handling, and adverse ventricular remodeling, providing a plausible biological link between inflammatory activation and worse HF trajectories [4,5,6].

In clinical practice and research, inflammatory biomarkers are attractive because they are accessible, scalable, and potentially actionable. High-sensitivity C-reactive protein (hs-CRP) is a prototypical acute-phase reactant integrating upstream cytokine signaling and has been repeatedly associated with HF incidence and adverse prognosis across EF phenotypes. Meta-analytic data in HFpEF support both diagnostic and prognostic relevance of CRP, while large contemporary cohorts of acute HF demonstrate independent associations between hs-CRP and long-term mortality and rehospitalization [7,8]. Alongside hs-CRP, interleukin-6 (IL-6) has emerged as a key mediator in HF biology, linking inflammatory activation to hepatic acute-phase response, myocardial hypertrophy/fibrosis, renal sodium handling, and adverse outcomes. In BIOSTAT-CHF, IL-6 carried clinical significance across a broad HF population, and additional long-term data suggest that IL-6 (with hs-CRP) may refine risk prediction particularly in HFpEF [6,9,10].

Nevertheless, the translation of inflammatory biomarkers into routine HF risk stratification remains inconsistent. Current international guidelines emphasize natriuretic peptides and high-sensitivity troponins for diagnosis and prognostication, while inflammatory biomarkers are less standardized due to variability in patient selection, timing of measurement (stable vs. decompensated; early hospitalization vs. convalescence), confounding by infection and comorbid inflammatory states, and uncertainty regarding incremental value over established clinical and cardiac biomarkers [2,3]. In hospitalized cohorts, this uncertainty is amplified: acute decompensation frequently coexists with systemic stress, renal dysfunction, congestion-driven organ injury, and occult infection, which may modulate inflammatory biomarker levels and their association with clinical outcomes. Procalcitonin-based work in dyspneic/acute HF populations further illustrates the complexity of interpreting inflammatory signals in real-world acute care settings [11].

Accordingly, there is a clinically meaningful rationale to evaluate inflammatory biomarkers specifically in hospitalized patients with pre-existing HF, where baseline vulnerability and comorbidity burden may magnify the prognostic relevance of inflammatory activation. In addition to hs-CRP and IL-6, emerging biomarkers reflecting inflammatory-fibrotic signaling (e.g., soluble ST2) or macrophage-driven fibrosis (e.g., galectin-3) may capture complementary biological axes and have shown prognostic or epidemiologic associations in HF [12,13,14]. A focused, single-center cohort analysis can therefore clarify biomarker–outcome relationships under consistent clinical workflows, laboratory timing, and outcome adjudication, while generating hypotheses for more advanced risk models and inflammation-targeted strategies.

In this context, the present single-center cohort study investigates the association between inflammatory biomarkers and clinically relevant outcomes in hospitalized patients with pre-existing HF, aiming to (i) characterize the inflammatory biomarker profile at/around hospitalization, and (ii) examine whether inflammatory biomarker levels are associated with adverse outcomes beyond conventional clinical risk factors, thereby informing more precise risk stratification in this high-risk population [1,2,3,4,5,6,8,9,10,11,12,13,14,15].

## 2. Materials and Methods

### 2.1. Study Design and Setting

This retrospective, single-center cohort study was conducted at the “Dr. Victor Babeș” Clinical Hospital of Infectious Diseases and Pneumophthisiology, a tertiary referral center in Timișoara, Romania. The hospital serves as a regional reference center for the management of severe infectious diseases, including coronavirus disease 2019 (COVID-19).

Consecutive adult patients admitted to the Department of Infectious Diseases I between March 2020 and December 2024 for confirmed COVID-19 were screened for eligibility. The study was designed to evaluate the prognostic significance of inflammatory biomarkers in hospitalized patients with pre-existing heart failure, within a real-world clinical setting.

### 2.2. Study Population

COVID-19 was diagnosed in accordance with national and institutional protocols, based on a positive reverse transcription polymerase chain reaction (RT-PCR) assay or a validated rapid antigen test detecting severe acute respiratory syndrome coronavirus 2 (SARS-CoV-2).

Eligible participants were adults (≥18 years) hospitalized with confirmed COVID-19 who had available baseline inflammatory and cardiac biomarker assessments obtained at hospital admission or within the first 24 h.

Exclusion criteria were: (1) incomplete documentation regarding pre-existing heart failure or other major comorbidities; (2) missing core biomarker data (C-reactive protein, interleukin-6, procalcitonin, D-dimer, NT-proBNP, or cardiac troponin); (3) missing data on primary outcomes (sepsis or mortality); (4) transfer from another hospital after a prolonged prior admission; (5) hospitalizations in which acute SARS-CoV-2 infection was not the primary reason for admission.

### 2.3. Data Collection and Clinical Variables

Electronic hospital medical records were reviewed to extract demographic, clinical, laboratory, and outcome data using a standardized data abstraction protocol.

Baseline variables included age, sex, body mass index (BMI), vaccination status against SARS-CoV-2, symptom onset timing, and vital signs at admission. Comorbidities were recorded based on documented medical history and included heart failure, ischemic heart disease, hypertension, diabetes mellitus, chronic kidney disease, chronic liver disease, chronic lung disease, obesity, and prior thromboembolic events.

COVID-19 severity at presentation was classified as mild, moderate, severe, or critical, based on respiratory status, oxygen requirements, and radiologic findings, according to contemporaneous national guidance and World Health Organization recommendations. Pulmonary involvement on chest imaging was categorized as absent, unilateral, or bilateral.

The in-hospital clinical course was documented, including: need for supplemental oxygen and delivery modality, admission to the intensive care unit (ICU), use and duration of invasive mechanical ventilation, development of sepsis or other complications (acute kidney injury, cardiovascular complications), length of hospital stay, and vital status at discharge.

### 2.4. Biomarker Assessment

All laboratory analyses were performed in the hospital’s accredited central laboratory using standardized methods and internal quality control procedures.

Complete blood counts were measured using automated hematology analyzers (Sysmex XN-1000, Sysmex Corporation, Kobe, Japan). C-reactive protein concentrations were determined by immunoturbidimetric assays (Cobas Integra 400 Plus, Roche Diagnostics, Mannheim, Germany). Interleukin-6 levels were measured using an electrochemiluminescence immunoassay (Elecsys IL-6, Cobas e601, Roche Diagnostics, Mannheim, Germany).

D-dimer concentrations were assessed using an immunoturbidimetric method (STA-Liatest D-Di, Diagnostica Stago, Asnières-sur-Seine, France) on the STA Compact Max analyzer. Procalcitonin levels were determined by chemiluminescent immunoassay according to the manufacturer’s instructions.

Cardiac biomarkers included N-terminal pro-B-type natriuretic peptide (NT-proBNP), reflecting myocardial wall stress, and high-sensitivity cardiac troponin, reflecting myocardial injury. Both biomarkers were measured using automated immunoassays in accordance with manufacturer specifications.

Additional laboratory parameters recorded included serum creatinine, urea, liver enzymes (aspartate aminotransferase, alanine aminotransferase), albumin, serum lactate, and arterial blood gas parameters (PaO_2_, PaCO_2_, pH, bicarbonate, and SaO_2_/FiO_2_ ratio).

### 2.5. Definition of Pre-Existing Heart Failure

Pre-existing heart failure was defined as a documented clinical diagnosis established prior to the index hospitalization, irrespective of left ventricular ejection fraction phenotype (reduced, mildly reduced, or preserved). Classification was based on cardiology records, prior heart failure hospitalizations, chronic use of guideline-directed medical therapy for heart failure, and diagnostic coding in the electronic medical record system. When available, International Classification of Diseases (ICD-10) codes for heart failure were also used to confirm the diagnosis.

When available, baseline NYHA functional class and echocardiographic parameters, including left ventricular ejection fraction and diastolic function, were abstracted. Echocardiographic data were available only in a subset of patients and were used descriptively; heart failure classification relied exclusively on pre-existing clinical documentation.

### 2.6. Outcomes

The primary outcomes of the study were: (1) development of sepsis during hospitalization, defined according to Sepsis-3 criteria as suspected or confirmed infection associated with an increase in Sequential Organ Failure Assessment (SOFA) score ≥ 2 points from baseline; and (2) all-cause in-hospital mortality.

Secondary outcomes included ICU admission, need for and duration of invasive mechanical ventilation, acute kidney injury, in-hospital cardiovascular complications (acute decompensated heart failure, clinically significant arrhythmias, acute coronary syndromes), and length of hospital stay.

### 2.7. Statistical Analysis

Continuous variables were assessed for distribution normality using the Shapiro–Wilk test and are presented as mean ± standard deviation or median with interquartile range (IQR), as appropriate. Categorical variables are summarized as counts and percentages.

Between-group comparisons were performed using Student’s *t*-test or Mann–Whitney U test for continuous variables and χ^2^ test or Fisher’s exact test for categorical variables, as appropriate. Due to right-skewed distributions, inflammatory biomarkers (CRP, IL-6, procalcitonin, and D-dimer) were analyzed using median (IQR) values and log-transformed for regression analyses when required.

Univariable logistic regression analyses were initially conducted to evaluate associations between pre-existing heart failure, inflammatory biomarkers, and study outcomes. Multivariable logistic regression models were subsequently constructed to identify independent predictors of sepsis and in-hospital mortality.

Covariates included in multivariable models were selected a priori based on clinical relevance and the existing literature and comprised age, sex, major comorbidities (hypertension, diabetes mellitus, chronic kidney disease), baseline COVID-19 severity, and key inflammatory and cardiac biomarkers (CRP, IL-6, procalcitonin, D-dimer, NT-proBNP, and troponin). Given the relatively limited number of outcome events, variable selection was intentionally restricted to clinically relevant predictors with established associations with COVID-19 severity or heart failure prognosis in prior studies. Model complexity was carefully limited to avoid overfitting, considering the number of outcome events.

To minimize the risk of model overfitting given the limited number of outcome events, the number of predictors included in the final multivariable mortality model was carefully restricted. The final model included eight prespecified covariates (age, sex, heart failure status, COVID-19 severity at admission, interleukin-6, C-reactive protein, NT-proBNP, and cardiac troponin), selected based on biological plausibility and prior literature. With 29 mortality events observed in the cohort, the event-per-variable ratio was approximately 3.6. Although this ratio is lower than the conventional rule-of-thumb threshold of 10 events per variable, recent methodological studies suggest that smaller ratios may still provide informative estimates when predictors are clinically justified and model complexity is limited.

Penalized regression techniques, such as Firth correction, were considered to address potential small-sample bias. However, given the exploratory nature of the study and the predefined clinical covariates included in the model, conventional multivariable logistic regression was retained to preserve interpretability and comparability with prior COVID-19 and heart failure cohort studies.

Multicollinearity was assessed using variance inflation factors. Model calibration was evaluated using the Hosmer–Lemeshow goodness-of-fit test, and discrimination was assessed using the area under the receiver operating characteristic curve (AUC).

Vaccination status was evaluated descriptively and was not included in multivariable models due to the limited number of outcome events.

Patients with missing data for variables included in a specific analysis were excluded from that analysis (complete-case approach).

Results are reported as odds ratios (ORs) with 95% confidence intervals (CIs). A two-sided *p* value < 0.05 was considered statistically significant.

All analyses were performed using IBM SPSS Statistics for Windows, version 26.0 (IBM Corp., Armonk, NY, USA).

### 2.8. Ethical Considerations

The study was conducted in accordance with the principles of the Declaration of Helsinki and complied with national and institutional regulations governing biomedical research. The research protocol was approved by the Ethics Committee of the “Dr. Victor Babeș” Clinical Hospital of Infectious Diseases and Pneumophthisiology, Timișoara, Romania (approval no. 11235/27.11.2025).

At hospital admission, all patients provided written informed consent for hospitalization and for the potential use of anonymized clinical data for research purposes, in accordance with institutional policy.

## 3. Results

### 3.1. Study Population and Baseline Outcomes

A total of 395 consecutive adult patients hospitalized with confirmed COVID-19 were included in the analysis, of whom 143 patients (36.2%) had pre-existing heart failure (HF), as shown in Table 1. Overall, in-hospital mortality was 7.3% (29 patients), and 5.3% (21 patients) required intensive care unit (ICU) admission. Vaccination against SARS-CoV-2 prior to hospitalization was documented in 262 patients (66.3%). Vaccinated patients were less frequent among those with pre-existing heart failure compared with patients without heart failure (58.0% vs. 71.0%, *p* = 0.012).

Patients with pre-existing HF had significantly higher in-hospital mortality compared with those without HF (11.9% vs. 4.8%, *p* = 0.016). ICU admission was also more frequent in the HF group (8.4% vs. 3.6%), showing a trend toward statistical significance (*p* = 0.069).

These findings indicate that pre-existing HF identifies a clinically vulnerable subgroup with substantially increased risk of adverse in-hospital outcomes.

### 3.2. Inflammatory Biomarkers at Admission According to Heart Failure Status

**Figure 1:**
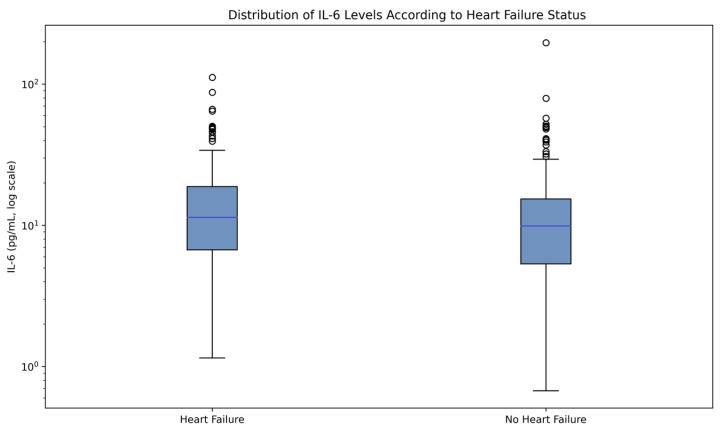
Distribution of admission interleukin-6 (IL-6) levels according to pre-existing heart failure status. Box-and-whisker plots illustrate IL-6 concentrations measured at hospital admission in patients with and without pre-existing heart failure. The central line represents the median, boxes indicate the interquartile range (IQR), and whiskers denote the range excluding outliers. Data are displayed on a logarithmic scale to account for right-skewed biomarker distribution. Patients with pre-existing heart failure exhibited higher IL-6 levels, reflecting enhanced systemic inflammatory activation during acute SARS-CoV-2 infection.

At hospital admission, patients with pre-existing HF exhibited a more pronounced inflammatory profile compared with patients without HF. Median IL-6 levels were numerically higher in the HF group (14.31 [4.62–43.75] vs. 9.49 [3.82–26.13] pg/mL, *p* = 0.055), although the difference did not reach conventional statistical significance, as illustrated in Figure 1, while CRP levels also showed a numerical increase (116.0 [61.0–152.4] vs. 105.5 [45.9–138.9] mg/L, *p* = 0.083), as in Table 2.

Procalcitonin and D-dimer concentrations were comparable between groups.

These findings suggest enhanced systemic inflammatory activation in patients with HF during acute SARS-CoV-2 infection.

Patients with HF showed higher systemic inflammatory activation, particularly reflected by IL-6, suggesting enhanced cytokine-mediated vulnerability during acute SARS-CoV-2 infection.

### 3.3. Heart Failure Status, Inflammation, and Mortality

Patients who died during hospitalization exhibited markedly elevated admission inflammatory biomarker levels, particularly CRP and IL-6.

In univariable logistic regression analysis, pre-existing HF was significantly associated with in-hospital mortality (OR 2.67, 95% CI 1.22–5.83, *p* = 0.014). Elevated IL-6 levels were also associated with mortality (OR per log-unit increase 1.45, 95% CI 1.12–1.89, *p* = 0.006).

**Figure 2:**
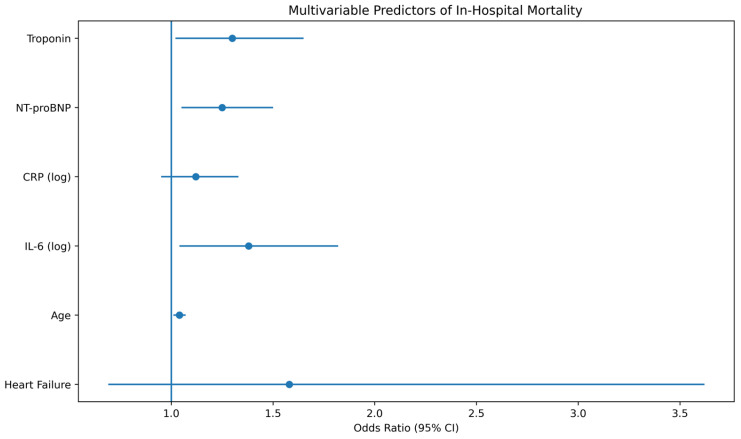
Multivariable predictors of in-hospital mortality. Forest plot showing adjusted odds ratios (ORs) with 95% confidence intervals (CIs) for variables included in the multivariable logistic regression model evaluating predictors of in-hospital mortality. The vertical reference line represents an OR of 1. Variables positioned to the right of the line indicate increased mortality risk. Variables included in the model were age, sex, heart failure status, COVID-19 severity, interleukin-6 (IL-6), C-reactive protein (CRP), N-terminal pro-B-type natriuretic peptide (NT-proBNP), and cardiac troponin. Interleukin-6 remained independently associated with mortality after adjustment for demographic factors, comorbidities, baseline disease severity, and cardiac biomarkers, supporting the role of systemic inflammatory activation as a key determinant of adverse outcomes. Blue dots represent the point estimates of the adjusted odds ratios, and the horizontal lines indicate the corresponding 95% confidence intervals.

In multivariable analysis adjusting for age, comorbidities, baseline disease severity, and inflammatory biomarkers, the independent association between HF and mortality was attenuated (adjusted OR 1.58, 95% CI 0.69–3.62, *p* = 0.28), whereas IL-6 remained independently associated with mortality risk (adjusted OR 1.38, 95% CI 1.04–1.82, *p* = 0.021). Independent predictors of in-hospital mortality are presented in Figure 2.

These findings suggest that systemic inflammatory activation partially mediates the excess mortality risk observed in patients with pre-existing HF.

### 3.4. Sepsis and Inflammatory Biomarkers

Development of sepsis during hospitalization was associated with significantly higher admission levels of IL-6 and procalcitonin. Patients with HF were more likely to develop sepsis, although this association weakened after adjustment for inflammatory biomarkers.

These findings support a model in which heightened inflammatory activation, rather than HF status alone, is the primary driver of organ dysfunction.

### 3.5. Secondary Outcomes

Patients with pre-existing HF experienced higher rates of ICU admission, increased need for invasive mechanical ventilation, and longer hospital stays compared with patients without HF.

**Figure 3:**
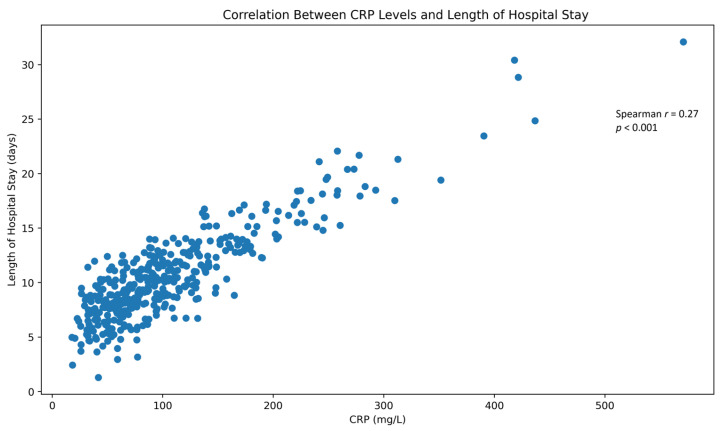
Association between admission C-reactive protein (CRP) levels and length of hospital stay. Scatter plot illustrating the relationship between CRP concentrations measured at hospital admission and duration of hospitalization. Higher CRP levels were associated with prolonged hospital stay (Spearman *r* = 0.27, *p* < 0.001), suggesting that systemic inflammatory burden influences both disease severity and recovery trajectory in hospitalized patients with COVID-19. Each point represents an individual patient.

Length of hospitalization correlated positively with admission CRP and IL-6 levels, indicating that inflammatory burden influences both disease severity and recovery trajectory. A positive association between CRP levels and length of hospital stay is illustrated in Figure 3.

## 4. Discussion

### 4.1. Principal Findings

In this single-center cohort of hospitalized patients with COVID-19, pre-existing heart failure (HF) identified a large and clinically vulnerable subgroup, accounting for more than one third of admissions. Patients with HF experienced a significantly higher in-hospital mortality and a greater need for intensive care, confirming HF as a major modifier of short-term prognosis in acute systemic illness. Importantly, HF patients also exhibited more pronounced inflammatory activation at admission, particularly reflected by higher interleukin-6 (IL-6) levels, supporting the concept that inflammatory burden partially mediates excess risk in this population.

While HF was strongly associated with mortality in unadjusted analyses, the attenuation of this association after inclusion of inflammatory biomarkers suggests that systemic inflammation acts as a key pathophysiological intermediary, rather than HF status alone being a purely structural risk marker. This finding refines the current understanding of vulnerability in HF patients hospitalized with COVID-19 and emphasizes the role of immune–cardiovascular crosstalk in driving adverse outcomes.

### 4.2. Heart Failure as an Inflammatory-Prone Clinical State

Chronic HF is increasingly recognized as a condition characterized by persistent low-grade inflammation, immune dysregulation, and endothelial activation. Elevated circulating cytokines, including IL-6 and tumor necrosis factor-α, have been documented even in clinically stable HF and are associated with disease progression and adverse outcomes [16,17,18]. Several mechanistic pathways may explain this predisposition, including gut barrier dysfunction with endotoxin translocation, neurohormonal activation, venous congestion–induced organ injury, and impaired resolution of inflammation [19,20].

In the setting of acute SARS-CoV-2 infection, this pre-existing inflammatory milieu may amplify host responses, leading to exaggerated cytokine release, endothelial dysfunction, and microvascular injury. Our observation that HF patients present with higher admission IL-6 levels supports this hypothesis and aligns with experimental data showing heightened cytokine responsiveness in failing myocardium and peripheral tissues [21].

### 4.3. IL-6 as a Central Mediator of Adverse Outcomes

Among all inflammatory biomarkers evaluated, IL-6 emerged as the most consistently associated marker with both mortality and sepsis. IL-6 occupies a central position at the intersection of innate immunity, hepatic acute-phase response, coagulation activation, and myocardial dysfunction. Beyond its role as a marker, IL-6 exerts direct biological effects on cardiomyocytes, promoting hypertrophy, impaired contractility, mitochondrial dysfunction, and fibrosis [22,23,24].

Clinical studies outside the COVID-19 setting have demonstrated that IL-6 predicts incident HF, HF hospitalization, and cardiovascular mortality in diverse populations [25,26]. In acute infectious states, elevated IL-6 reflects both pathogen-driven immune activation and host vulnerability, providing a plausible explanation for its strong prognostic signal in HF patients hospitalized with COVID-19. Our findings therefore support IL-6 as a pathophysiologically relevant and clinically informative biomarker, rather than a nonspecific inflammatory bystander.

Systemic inflammation may also have important electrophysiological consequences that contribute to adverse cardiovascular outcomes in patients with COVID-19. Increasing evidence suggests that inflammatory cytokines, particularly interleukin-6, may promote arrhythmogenesis through multiple mechanisms [27]. Cytokine-mediated myocardial injury can alter cardiomyocyte electrophysiological properties and promote electrical instability. In addition, inflammatory signaling may directly modulate cardiac ion channels, affecting repolarization and conduction velocity. Microvascular dysfunction and endothelial activation—frequently observed during severe systemic inflammation—may further impair myocardial perfusion and exacerbate electrical heterogeneity. Finally, inflammatory activation may contribute to autonomic imbalance with increased sympathetic activity, which represents a recognized trigger for atrial and ventricular arrhythmias. In this context, elevated IL-6 levels observed in our cohort may not only reflect systemic inflammatory burden but may also contribute to electrical vulnerability, potentially increasing the risk of arrhythmic complications in patients with pre-existing heart failure hospitalized with COVID-19.

### 4.4. Systemic Inflammation, Sepsis, and Multiorgan Dysfunction

The higher incidence of sepsis among HF patients and its strong association with inflammatory biomarkers underscore the susceptibility of this population to infection-related organ dysfunction. HF-related reductions in cardiac reserve, renal perfusion, and microcirculatory adaptability may lower the threshold for acute organ failure once systemic inflammation is triggered [28,29].

Importantly, after adjustment for IL-6 and procalcitonin, the independent contribution of HF to sepsis risk was attenuated, suggesting that biomarker-defined inflammatory burden captures much of the excess risk traditionally attributed to HF alone. This observation is consistent with the recent sepsis literature emphasizing host-response phenotypes over comorbidity labels as drivers of outcome heterogeneity [30,31].

### 4.5. Clinical Implications

From a clinical perspective, our findings have several important implications. First, HF patients hospitalized with COVID-19 should be recognized as a high-risk inflammatory phenotype, even at early stages of admission. Second, routinely available inflammatory biomarkers—particularly IL-6—may enhance early risk stratification beyond conventional clinical assessment. Third, the partial mediation of HF-associated risk by inflammation suggests that targeting inflammatory pathways may provide a complementary therapeutic approach to purely hemodynamic optimization in selected patients.

Although anti–IL-6 therapies have shown mixed results in unselected COVID-19 populations, it is plausible that biomarker-guided, phenotype-specific approaches may yield greater benefit in patients with underlying cardiovascular vulnerability [32,33,34]. Previous clinical investigations have similarly highlighted the prognostic relevance of inflammatory and cardiovascular biomarkers in hospitalized COVID-19 populations, supporting the concept that biomarker-guided risk stratification may improve clinical decision-making in patients with underlying cardiovascular disease [35]. Future studies should explore whether inflammatory marker–based stratification can inform tailored therapeutic strategies in HF patients during acute systemic illness.

From a precision-medicine perspective, biomarker-guided identification of inflammatory phenotypes may represent a pragmatic strategy to improve risk stratification in patients with underlying cardiovascular vulnerability during acute systemic illness.

Beyond inflammation-targeted therapies, it is also important to consider the potential influence of contemporary guideline-directed heart failure treatments on inflammatory pathways and clinical outcomes in patients with COVID-19. Sodium–glucose cotransporter-2 (SGLT2) inhibitors, including empagliflozin and dapagliflozin, have demonstrated pleiotropic effects extending beyond their hemodynamic and metabolic benefits. Experimental and clinical studies suggest that these agents exert anti-inflammatory, endothelial-protective, and anti-arrhythmic effects, which may contribute to improved cardiovascular resilience during systemic inflammatory states such as acute viral infections.

In addition, vericiguat, a soluble guanylate cyclase stimulator targeting the nitric oxide–sGC–cyclic guanosine monophosphate (cGMP) signaling pathway, represents another therapeutic approach closely linked to endothelial dysfunction and inflammatory activation in heart failure. By enhancing NO–sGC–cGMP signaling, vericiguat may improve vascular function and myocardial performance in conditions characterized by impaired endothelial signaling and systemic inflammation. Recent position papers have highlighted the relevance of this pathway in the pathophysiology and management of heart failure, suggesting that therapies modulating endothelial and inflammatory signaling may play an important role in high-risk populations with overlapping cardiovascular and infectious stressors [36].

The broader context of heart failure management during the COVID-19 pandemic should also be considered when interpreting the present findings. During pandemic waves, healthcare systems faced substantial pressure, which accelerated the implementation of remote monitoring and home-based management strategies for patients with chronic cardiovascular disease. In particular, telemedicine approaches and remote surveillance of patients with implanted cardiac devices (including pacemakers, implantable cardioverter-defibrillators, and cardiac resynchronization therapy systems) have enabled earlier detection of clinical deterioration, arrhythmic events, and fluid overload while reducing unnecessary hospital visits. These strategies have been shown to help maintain continuity of care, optimize risk stratification, and reduce hospital burden in vulnerable heart failure populations during pandemic conditions [37]. In this context, integrating biomarker-based risk stratification—such as inflammatory markers identified in the present study—with remote monitoring approaches may represent a promising strategy to improve management of high-risk HF patients during future healthcare disruptions.

### 4.6. Strengths and Limitations

The strengths of this study include a well-characterized real-world cohort, systematic biomarker assessment at admission, and clinically meaningful outcomes. The focus on pre-existing HF in a hospitalized COVID-19 population addresses an important evidence gap.

Several limitations merit consideration. The single-center, retrospective design limits causal inference and generalizability. Heart failure classification relied on documented clinical history rather than systematic echocardiographic phenotyping, as comprehensive echocardiographic data were available only for a subset of patients. Consequently, we were unable to uniformly classify patients according to HF phenotypes (HFrEF, HFmrEF, HFpEF). This limitation reflects the retrospective nature of the study and routine clinical documentation during the pandemic period but may have introduced heterogeneity within the HF group. Inflammatory biomarkers were assessed at admission only, precluding evaluation of dynamic changes. Finally, residual confounding cannot be fully excluded despite multivariable adjustment. Additionally, the relatively small number of outcome events (29 in-hospital deaths and 21 ICU admissions) may have limited statistical power for extensive multivariable adjustment and for detecting subtle associations, particularly for marginal differences (e.g., admission IL-6 and CRP levels between groups). The relatively limited number of mortality events may also have constrained the stability of multivariable estimates, and therefore the adjusted models should be interpreted as exploratory rather than definitive. This limitation also raises the possibility of model overfitting despite efforts to restrict model complexity. Being a retrospective study, it was not prospectively powered to detect specific effect sizes, which increases the risk of type II errors and may explain why some univariable associations attenuated after adjustment. The present analysis was designed to explore associations between inflammatory biomarkers and clinical outcomes rather than to develop a formal predictive model. Therefore, diagnostic performance metrics such as sensitivity, specificity, and optimal prediction thresholds were not calculated. Future studies with larger multicenter cohorts may enable the development and validation of clinically applicable prediction models based on inflammatory biomarkers.

Another limitation relates to the extended enrollment period (2020–2024), which spans multiple phases of the COVID-19 pandemic. Vaccination status also differed between patients with and without heart failure. Although vaccination was recorded and reported descriptively, it was not included in multivariable models due to the limited number of outcome events and the need to restrict model complexity. Vaccination may nevertheless have influenced disease severity and clinical outcomes, representing a potential residual confounder that could not be fully addressed in the present analysis. During this time, circulating viral variants, treatment strategies, and population vaccination coverage evolved substantially. These temporal changes may have influenced disease severity, inflammatory responses, and clinical outcomes, potentially introducing heterogeneity within the study cohort. Although vaccination status was recorded descriptively, the retrospective design and limited number of outcome events precluded detailed temporal or variant-specific analyses.

## 5. Conclusions

In hospitalized patients with COVID-19, pre-existing heart failure identifies a population at substantially increased risk of adverse clinical outcomes, including in-hospital mortality and sepsis. This excess risk appears to be partly mediated by heightened systemic inflammatory activation, with interleukin-6 emerging as a key biomarker linking cardiovascular vulnerability, immune dysregulation, and organ dysfunction. These findings support a pathophysiological framework in which inflammation-sensitive risk stratification may complement traditional cardiovascular assessment and inform more personalized management strategies in high-risk patients. Prospective multicenter studies are warranted to validate these observations and explore biomarker-guided therapeutic approaches.

## Figures and Tables

**Table 1:** In-hospital outcomes according to heart failure status.

Outcome	Total (*n* = 395)	HF (*n* = 143)	No HF (*n* = 252)	*p* Value
In-hospital mortality, *n* (%)	29 (7.3)	17 (11.9)	12 (4.8)	0.016
ICU admission, *n* (%)	21 (5.3)	12 (8.4)	9 (3.6)	0.069
Vaccinated against SARS-CoV-2, *n* (%)	262 (66.3)	83 (58.0)	179 (71.0)	0.012

**Table 2:** Admission inflammatory biomarkers according to heart failure status.

Biomarker	HF (Median, IQR)	No HF (Median, IQR)	*p* Value
CRP (mg/L)	116.0 (61.0–152.4)	105.5 (45.9–138.9)	0.083
IL-6 (pg/mL)	14.31 (4.62–43.75)	9.49 (3.82–26.13)	0.055
Procalcitonin (ng/mL)	0.39 (0.15–0.90)	0.45 (0.14–0.90)	0.714
D-dimer (mg/L)	0.77 (0.47–1.27)	0.74 (0.52–0.99)	0.451

## Data Availability

De-identified clinical, laboratory, and imaging data supporting the findings of this study are available from the corresponding authors upon reasonable request, subject to institutional data-sharing policies.

## Abbreviations

The following abbreviations are used in this manuscript:

AKI	Acute Kidney Injury
AUC	Area Under the Curve
BMI	Body Mass Index
CI	Confidence Interval
COVID-19	Coronavirus Disease 2019
CRP	C-Reactive Protein
HF	Heart Failure
HFmrEF	Heart Failure with Mildly Reduced Ejection Fraction
HFpEF	Heart Failure with Preserved Ejection Fraction
HFrEF	Heart Failure with Reduced Ejection Fraction
hs-CRP	High-Sensitivity C-Reactive Protein
ICU	Intensive Care Unit
IL-6	Interleukin-6
IQR	Interquartile Range
NT-proBNP	N-Terminal Pro-B-Type Natriuretic Peptide
NYHA	New York Heart Association
OR	Odds Ratio
PaCO_2_	Partial Pressure of Carbon Dioxide
PaO_2_	Partial Pressure of Oxygen
RT-PCR	Reverse Transcription Polymerase Chain Reaction
SaO_2_	Arterial Oxygen Saturation
SOFA	Sequential Organ Failure Assessment
SARS-CoV-2	Severe Acute Respiratory Syndrome Coronavirus 2
